# Montmorillonite in next-generation drug delivery: patent trends, safety insights, and industrial advancements

**DOI:** 10.1007/s10856-026-07031-4

**Published:** 2026-04-24

**Authors:** Afzal Haq Asif, B. R. Asha, Samathoti Prasanthi, Prakash Goudanavar, Kalappa Prashantha, K. S. Srikruthi, N. Raghavendra Naveen, Girish Meravanige, Predeepkumar Narayanappa Shiroorka, Krishna Swaroop, Pavan Kumar Pavagada Sreenivasalu, Nagaraja Sreeharsha

**Affiliations:** 1 Department of Pharmacy Practice, College of Clinical Pharmacy, King Faisal University, Al-Ahsa, Saudi Arabia; 2 Department of Pharmaceutics, Sri Adichunchanagiri College of Pharmacy, Adichunchanagiri University, Karnataka, India; 3 Department of Pharmaceutics, MB School of Pharmaceutical Sciences (Erstwhile Sree vidyanikethan College of Pharmacy), Mohan Babu University, Sree Sainathnagar, Tirupati, Andhra Pradesh India; 4 Centre for Research and Innovation, Adichunchanagiri University, Karnataka, India; 5 Department of Biomedical Sciences, College of Medicine, King Faisal University, Al-Ahsa, Saudi Arabia; 6 Department of Restorative Dental Sciences, College of Dentistry, King Faisal University, Al-Ahsa, Saudi Arabia; 7 Department of Pharmaceutical Sciences, College of Clinical Pharmacy, King Faisal University, Al-Ahsa, Saudi Arabia

## Abstract

Montmorillonite (MMT) is a natural clay mineral and the major component of bentonite, and it possesses an exceptional swelling capacity, a high potential for adsorption, and excellent biocompatibility. Therefore, MMT has been identified as an attractive candidate for pharmaceutical applications in drug nano delivery systems. In recent years, research on MMT has also gained momentum because of its ability to improve the solubility, rate of dissolution, and bioavailability of poor-water-soluble drugs in polymeric nanocomposites. Although much research has been done on the formulation advantages of MMT, critical analysis of their translational progress and industrial uptake remains inadequate. This reviews an in-depth study of the role of MMT as a pharmaceutical excipient in nano drug delivery through a synergy of published work and patent landscape analysis. Patents on MMT-based formulations are sequenced and scrutinized to identify significant players in the industry, therapeutic applications, and emerging patterns of technology. In the comparative assessment of research publications vis-a-vis patents in the decade under review, evolving innovation patterns have of late drawn attention to scalable, multifunctional, clinically relevant MMT nanocomposites. Overall, the review indicates how MMT is gaining importance in pharmaceutical development and what it will provide for the future concerning rational design, regulatory convergence, and commercialization of MMT-embedded polymeric nano drug delivery systems.

**Graphical Abstract:**

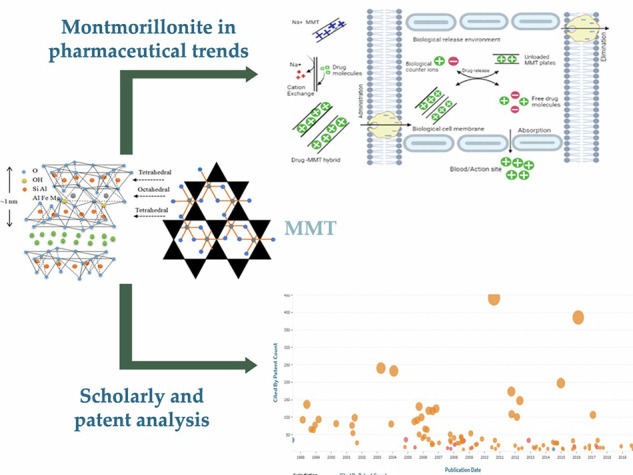

## Introduction

Since no one technique or field of science falls under the umbrella of nanotechnology, the term might be deceptive [1]. Instead, nanotechnology is a collection of procedures, substances, uses, and ideas categorized by size [2]. Nanotechnology works with materials that are between 1 and 100 nanometers in size, and as we will see in a moment, the physical properties of a given material (e.g. carbon, silicon, metals, etc.) on the nanoscale are different from those on the macroscale. The widespread use of nanotechnology in biomedicine and pharmaceuticals has the potential to change the way many drugs are taken [3, 4]. Nanocomposites are among the nanocarriers that have been approved for use in clinical applications. Heterogeneous, multicomponent types of nanocomposites with multiphase domains [4]. Due to their superior interfacial activity and drug absorption, polymer-based nanoclay composites are recognized as suitable carriers for the development of numerous drug delivery systems.

In the early 1980s, Roy, Komarneni, and their colleagues noted the non-uniformity of materials formed from sols and gels at the nanoscale, leading to the first use of the term “nanocomposite” in connection with sol-gel processes. Nanocomposites refer to solids composed of two or more nanoscale regions that vary in structure and/or composition. The term appeared in the literature for the first time in a 1986 publication, and at that point, a Web of Science search revealed it as the sole reference to “nanocomposite.” Since then, its usage has grown exponentially, with nearly 59,400 references by the end of 2022. Today, a heterogeneous material is considered a nanocomposite when at least one of its components has a size ranging from a few angstroms to several nanometers [5].

Nanocomposites can be processed using the same methods that are used for new polymers, e.g. extrusion, injection molding, thermoforming, blow molding, compression molding or transfer molding [6]. Another advantage of these processes is the high throughput required for cost-effective production. For example, polymer or glass fiber reinforced automotive parts must be produced at a rate of more than one part per minute to compete with conventional materials and processes [6].

Nanocomposites come in various forms, including metal nanocomposites, polymer nanocomposites, and bio-nanocomposites. Organic or inorganic fillers that are uniformally disseminated inside a polymer matrix serve as additives in polymer nanocomposites. Biopolymers (bio-based and biodegradable) have gained popularity as polymer matrices in recent years [7]. The so-called polymer nanocomposites that have an organic-inorganic structure are materials that contain at least one phase of filler with a size less than 100 nm [7]. Various approaches are used to develop polymer nanocomposites. However, four traditional synthesis modes are most widely used: Template synthesis, enamel intercalation, in situ polymerization intercalation and exfoliation adsorption [8]. Typically, materials with fillers below 100 nm exhibit various microstructures on the basis of the techniques used for their production template synthesis, enamel intercalation, in situ polymerization intercalation, and exfoliation adsorption. Non-intercalated microcomposite involves fillers distributed without substantial molecular bonding between them. Such type of composition only offers limited improvements in the property of polymer matrix. The intercalated structures, on the other hand, place fillers in between the polymer layers, resulting in better interaction and enhanced mechanical and thermal properties. The most superior performance among all types is provided by the exfoliation or nanolayer-separated nanocomposite, wherein the fillers are kept privately separated as individual sheets descend to the nanoscale in the polymer matrix. This gave better advantage in property when observed in mechanical strength, thermal stability, and barrier properties. Each microstructure type has some advantages that apply to certain application requirements [9].

Phyllosilicates are layered silicate minerals having a distinct two-dimensional sheet-like structure, which is characterized by high surface area and significant ion exchange ability. Thus, they confer advantages in soil science, material science, and drug delivery applications. Clays like kaolinite and montmorillonite are some examples of phyllosilicates with a wider range of applications. These phyllosilicates, a sub-group of silicate minerals, have a tetrahedral and octahedral arrangement forming a characteristic layered lattice. The localized arrangement along with intercalation ability makes phyllosilicates essential in various areas such as nanotechnology and drug delivery systems. Their versatility has made an important area of study in material science and environmental applications.

The existing studies have been quite fragmented and largely centered around material synthesis, physicochemical characterization, or isolated biomedical applications, especially when polymer nanocomposites and clay-based nanocarriers are concerned. In particular, altough drug delivery, wound healing, and biomaterial scaffolds have been the focus of much interest regarding MMT-based polymer nanocomposites, an integrated analysis directly and critically correlating material design strategy, structure-property relationships, translational potential, and industrial applicability is still elusive. Furthermore, the vast majority of review articles published so far have focused squarely on academia and have not taken into consideration the patent landscape, which is of paramount importance in the assessment of the technological maturity, trends in commercialization, and unmet challenges in formulation of the particular technology. This creates a clear knowledge disjunction among academic innovations and product realization.

In view of the above, the specific problem considered in this review is the absence of a well-defined flow of work that would visualize the linkage of polymer-clay nanocomposite sciences with patented technologies and translational outcomes. Without such an integration, it will be difficult to identify real innovation, avert duplication in formulation approaches, thereby directing future work toward clinically and commercially viable nanocomposite systems.

Thus, this review aims at a systematic investigation on polymer-nanoclay composites, particularly montmorillonite-based systems, bridging together research literature and patent evidence to forge a comprehensive picture considering the material composition, synthesis strategies, structure-property relationship, and performance driven by applications. Proposed here is an innovative join of peer-reviewed studies and patent analysis as avenues for exploring nascent trends, technological voids, and translational opportunities that taken together would not so evidently present themselves from an academic standpoint. This dual perspective will assist in a rational design, scaling, and regulatory alignment of the next-generation nanocomposite drug delivery system.

## Clay materials

Clay minerals make up most of the starting materials for clay production (clay rock). Except in a few cases, most of them have a crystal structure characterized by densely piled-up sheets that form structural layers (hence the name phyllosilicates) [10]. Hydrophilic clays can be easily functionalized and modified for a wider circumference of applications via ion exchange intercalation, impregnation with metal or metal complexes, columnarization, or acid treatment [Table 1]. A second type of clay has a more platelet-like structure; it’s usually less than 1 nm thick.

**Table 1:** Major groups of clay minerals

S. No	Group name	Member minerals	Composition	Application
1.	Kaolinite	Nacrite, Kaolinite and Dickite	Primarily aluminum silicate with a general formula of Al2Si2O5(OH)4.	Used in ceramics, paper coating, rubber, and as a filler in paints and plastics.
2.	Smectite	MMT, Vermiculite, Saponite, Pyrophyllite, Sauconite and nontronite.	Generally hydrated aluminum silicate with varying metal and structural components.	Utilized in drilling fluids, as an adsorbent, in waste treatment, and in the production of catalysts.
3.	Illite	Illite	A group of aluminosilicate minerals with a general formula of (K,H3O)Al2(Si,Al)4O10(OH)2·nH2O.	Commonly used in ceramics, as a filler in paints and rubber, and in soil conditioning.
4.	Chloride	Cookeite, Amesite, Chamosite and Nimite etc.	Typically contains a complex mixture of aluminum, magnesium, and iron silicates, with varying layers.	Used in the manufacture of refractories, as a filler in paints, and in some applications related to catalysis and environmental remediation.

### Clays classification

**Fig. 1:**
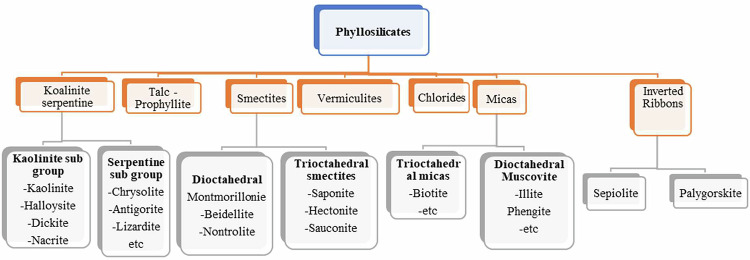
Phyllosilicate categorization

There are two classification systems for clays: (i) Depending on their origin, clays can be classified as exogenous (fluvial, weathered and sedimentary) or endogenous (intrinsically occurring) or (pneumatolytic or hydrothermal). ii) Based on structural chemistry [11]. Clays can be categorized into two primary groups based on their octahedral layer structure: dioctahedral (gibbsite-type layers) and trioctahedral (brucite-type layers). They are further divided into two-layer and three-layer structures, depending on whether the octahedral layer is linked to one or both sides of a tetrahedral layer. Two-layer structures include minerals like kaolinite, nacrite, dickite, halloysite (Al4Si4O10(OH)8), antigorite (platy serpentine), and chrysotile (fibrous serpentine) with the formula Mg6Si4O10(OH)8. Three-layer structures are represented by smectite montmorillonite (MMT) (Al2Si4O10(OH)2), muscovite (KAl2(AlSi13O10(OH)2), margarite (CaAl2(Al2Si2O10(OH)2), and talc (Mg3Si4O10(OH)2). Vermiculite (Mg3Si4O10) is another example [Fig. 1].

Bailey and Reider et al. classified clays into seven phyllosilicate groups: kaolinite-serpentine, talc-pyrophyllite, smectite, vermiculite, chlorite, mica, and the inverted-banded group. The kaolinite subgroup is described by the formula Al4Si4O10(OH)8, featuring alternating Si4O10 and gibbsite-like layers. Smectite group clays, which have pyrophyllite-like structures, swell in water due to exchangeable cations and varying water molecules between their layers. Fine-grained muscovite, known as Tong mica, often coexists with MMT. Chlorite, frequently found with other clay minerals, is challenging to isolate. Allophane clays, identified through X-ray diffraction, are the amorphous components in clay materials [12] [Table 2].

**Table 2:** Layerred structures of clay minerals

S. No	Clay mineral group	Layer type	Layer charge
1	Kaolinite	1:1	<0.01
2	MMT	2:1	0.5–1.2
3	Illite	2:1	1.4–2.0
4	Vermiculite	2:1	1.2–1.8
5	Chloride	2:1:1	Variable

## Clays’ ion exchange capacity

A key property of clays is their ion exchange capacity, which allows them to retain certain anions and cations in an exchangeable state after adsorption. These adsorbed ions can then be replaced by other anions or cations in an aqueous solution, though the exchange reaction can also occur in non-aqueous environments. This process typically has minimal impact on the structure of the silica-alumina framework, as the exchangeable ions are located near the exterior of the silica-alumina mineral’s structural units. A common example of ion exchange is water softening using zeolites, permutites, or carbon exchangers, where the ion exchange capacity is measured in milliequivalents per gram (meq/g). One meq per 100 grams equates to 0.031% Na2O, meaning one equivalent of sodium, expressed as Na2O, weighs 31 grams. Ion exchange capacities are generally calculated at a neutral pH of 7.

Due to their excellent properties, including high biocompatibility, nanoclay materials are increasingly used in the pharmaceutical, cosmetic, biomedical and medical industries for biological or therapeutic purposes and to ensure controlled drug release [13, 14]. Hydrotalcite and octasilicate have limitations in terms of their physical properties and cost. MMT, mica fluoride and hydrotalcite have no such limitations. While MMT is a naturally occurring clay, mica fluoride is synthetic. The MMT clays have the highest tolerance for use in pharmaceuticals and polymer nanocomposites and can be considered safe for many applications [15]. The following sections, therefore, deal with various aspects of MMT.

## MMT as a drug carrier

Montmorillonite (MMT) is a naturally found clay mineral and is described by having layered structures, much internal surface area, having high adsorption capacity and being least toxic [16, 17]. Such properties render this MMT very interesting to apply in pharmaceutical application, especially in drug delivery Nano-system. The MMT net negative charge gives good swelling in water and in any hydrophilic solvent. Hence, it would swell by intercalating positively charged bioactive compounds within its inter-layer spaces, following the electrostatic interactions [18]. Such interactions at a molecular level would give efficient loading of the drug into the structure without losing the structural integrity of the carrier. According to several studies conducted in the previous years, MMT has been a leading component of drug delivery systems that meet the most important pharmaceutical challenges such as poor aqueous solubility and uncontrolled release of the drug [19].

Incorporation of drugs into montmorillonite matrices improves dissolution behavior, control of release kinetics and enhanced bioavailability of poorly soluble therapeutic agents. MMT-based dosage forms, therefore, represent a very promising approach in the controlling of drug release profiles in the future and the improvement of therapeutic efficiency. Recent developments place montmorillonite as a versatile and promising nanocarrier contributing to the formulation of more effective and patient-compliant pharmaceutical delivery systems.

### MMT polymers in nanotechnology

MMT polymers have gained importance in nanotechnology due to their invaluable properties, which are transforming many applications. The layered silicate structure of MMT permits exfoliation into nanoscale sheets, thus significantly augmenting the surface area and mechanical strength. This makes it very effective in reinforcing nanocomposites in MMT polymers, increasing their mechanical strength, thermal stability, and barriers. MMT polymers are primarily used to formulate advanced nanocomposites for packaging and coatings. These types of materials are manufactured to increase the life and performance of such products against environmental conditions. Besides that, MMT has also great potential in intercalating organic molecules to form new materials for specific property requirements in the industry. MMT polymers in the pharmaceutical industry are then used as controlled and sustained delivery systems for active compounds in drug delivery systems. MMT high surface area and layered nature are suitable for encapsulation and release of drugs using this type of system. Thus, bioavailability and therapeutic efficacy of the medicines increase even further. From these very different examples, it will soon become apparent that MMT polymers have a marked influence on the development of nanotechnology in various fields.

## MMT properties

### Physical properties

Montmorillonite (MMT) possesses observable structures of layers: an octahedral sheet sandwiched in between two tetrahedral ones. Tetrahedral sheets are made up of a number of silicon-oxygen tetrahedra set in hexagonal arrangements while the octahedral sheet consists mainly of aluminum or magnesium coordinated with hydroxyl and oxygen groups. Tetrahedral-octaheral-tetrahedral units can be taken as a layer, however, several layers in a single clay crystallite are held together by cations with van der Waals forces, electrostatic interactions, and hydrogen bonding. Softness, color, hygroscopicity, texture, swelling behavior, etc. are a result of the physicochemical properties of this mineral, which is mainly due to the structural change occurring by isomorphous cation substitution within the tetrahedral and octahedral sheets. This type of substitution sets up a net negative charge and ultimately mineralogical diversity without having a fixed theoretical composition with completeness in nature because interlayer water molecules are always present (Table 3). Natural montmorillonite, as a rule, is silicoaluminous, i.e., the main structural components are silicon and aluminum: silicon is in tetrahedral coordination and aluminum in octahedral one. In all, chemical composition, layered structure, ionic substitutions, and particle size jointly determine the classification and functional properties of clay minerals.

**Table 3:** Few physical properties of MMT

S. No	Property name	Description
1	Density	2–3 g /cm^3^(measured)
2	Crystal system	Monoclinic
3	Hardness	1–2 on Mohs scale, soft, possess fine grained occurrence
4	Fracture	Irregular, uneven
5	Cleavage	Perfect
6	Luster	Earthy, dull
7	Transparency	Translucent
8	Color	White, buff, yellow, green, rarely pale pink to red.

### Chemical characteristics

Montmorillonite (MMT) is one of the most researched clays for polymer nanocomposite applications over the past three decades. MMT is a smectite clay with a 2:1 structure, which consists of two tetrahedral silica sheets surrounding a central octahedral aluminum oxide layer. Its chemical formula can be represented as Mx(Al4-xMgx)Si8O20(OH)4, where the variable “x” denotes the degree of isomorphous substitution (~0.5–1.3), and “M” denotes a monovalent cation. Due to its hydrophilic character, MMT shows poor compatibility with most organic polymers and, hence, has to be modified to ensure good dispersion of the clay into the polymer matrix. It is functionalized, aided by the hydroxyl groups present in its chemical structure, by means of grafting onto polymer chains.

The interlayer space of MMT also permits the adsorption and exchange of cations such as Na+ and K+. The cation exchange capacity (CEC) of MMT falls in the range of 80–100 mEq/100 g, which refers to the total amount of exchangeable cations per unit mass or surface area of the sample. This property is used in cation exchange routes to modify MMT with organic compounds.

### Functional characteristics

#### Cation exchange-related property

The cation exchange capacity (CEC) of a soil is influenced by its clay and organic matter contents. In terms of cation-holding capacity, it expresses the ability of soil to hold cations such as Al3+, Ca2+, Mg2+, Mn2+, Zn2+, Cu2+, Fe2+, Na+, K+, and H+ on the negatively charged surfaces of clay minerals. CEC is measured in centimoles of positive charge per kilogram of soil, or in milliequivalents (meq) per gram of soil. Thus, CEC indicates the capacity of soil to retain those essential nutrients, sometimes referred to as cation exchange potential [20]. Finer fractions of clay typically exhibit a greater ratio of surface area to unit weight as their diameter is between 0.002 and 0.001 mm. So, a surface area that is larger, capable of absorbing many more cations, brings about an increase in electrical conductivity to the clay material [21]. Ions of smaller size, such as Fe3+ and Al3+, may replace Si4+ in the tetrahedral coordination of sheet structure in the substitution of ions. Furthermore, Al3+ could be replaced in octahedral sheets by cations such as Li+, Ni2+, Cu2+, Mg2+, Fe2+, and Fe3+. Larger interlayer cations such as K+, Na+, and Cs+ are placed within the layers, changing the properties of clay [22].

#### Electrical conductivity

Porous materials that include clay particles experience changes in electrical conductivity due to the influence of pore fluid. The overall electrical conductivity expressed in mS/m results from the combined conductivities of the pore fluid and the matrix material. Pores can be filled with saline water, air, or plain water. When the pore fluid is of low conductivity, such as air or water, matrix materials act to assist in the boosting of the bulk conductivity of the clay particles [23]. Conversely, when saline water with a high conductivity is present as pore fluid, the contribution of the pore fluid to overall conductivity can become significant, especially when clay particles exhibit high porosity (40-50%). Under this scenario, the conductivity contrast between clay and sand becomes minimal. More clay particles with a 2:1 structure give higher bulk electrical conductivity of non-saline soils. Such conductivity is often associated with either exchangeable cations or proton transfer due to the dissolution of interlayer water content. Clays that are high in potassium (K) saturation and have low interlayer water content tend to have low electrical conductivity [24]. If the clay content and type, saline water or otherwise, and water saturation would affect soil conductivity, then the electrical properties of reservoir rocks would be an important area to study. Among other factors, variations in the distribution of liquid and solid phases are making the electrical conductivity of heterogeneous porous media difficult. Ions in electrical clays form a membrane in which ions are mainly polarized. When direct current is applied across the clay pore, the negative ions drift in one direction. This produces membrane polarization of the clay, treating the current to flow. In soil science, conductivity and membrane polarization have been employed to estimate the clay content [25].

#### Heat resistance

Being an excellent heat insulator, Montmorillonite (MMT) forms a great additive to various formulations concerning imparting heat resistance features. The ongoing research aims for the development of advanced heat-resistant materials from its thermal barrier properties. Clay minerals are increasingly being applied in fields that require heat and flame resistance due to their thermal barrier properties. Nanoclay, in particular, has gained considerable attention and is being investigated and utilized in polymer composites to improve their thermal stability and flame resistance. Different materials also expand thermally differently under heating. In general, ceramics are the most, followed by metals, while polymers come last. As for the reasoning behind this order: ceramics typically expand in the range of 20–100 ppm/°C, metals from 3 to 20 ppm/°C, and polymers from 3 to 5 ppm/°C [26]. MMT being a filler with good thermal stability can thus provide polymers with low thermal expansion. Polymer needs thermal stability improvement where aspect ratio above 100 is beneficial.

#### Water solubility sorption

Water sorption is an important property of natural clay minerals, and the clay particles will take in or lose water depending on changes in the surrounding relative humidity. In montmorillonite (MMT), absorbed water goes into the interlayer spaces between stacked silicate layers, causing swelling, while drying out will create a relatively stable non-expandable condition [27]. The extent of swelling is dependent on clay type, its mineral composition, and the nature of the accompanying phases, such as carbonates, feldspar, micas, and quartz. The process of swelling in MMT entails some complicated water-molecule-clay-platelet interaction mechanisms, where water state becomes hydrated and has hysteresis effects as a result of swelling. Redistribution of interlayer ions from surface sites toward the interlayer plane promotes expansion and signifies the importance of charge distribution on swelling [28]. Montmorillonite is a 2:1 clay mineral made of two tetrahedral sheets separated by an octahedral sheet, where isomorphous substitution of Si by Al or Al by Mg generates a net negative charge on the platelets.

This charge is balanced against interlayer cations, and hydration of the interlayer cations directly controls the dynamics of swelling. Monovalent (K⁺; Na⁺) and divalent (Ca²⁺) cations interact differently with water and MMT layers. Molecular dynamic simulations have proven highly useful in deciphering swelling mechanisms at the atomic level, where cation valence has been shown to greatly influence MMT-water interactions. K⁺ favors dehydrated sheets, whereas Ca²⁺ has a stronger affinity for hydrated layers. Experimental techniques measuring swelling in conjunction with pressure, FTIR, ATR, and SEM analyses have also indicated a gradually increasing misorientation of platelets with descending particle size and ascending swelling, especially in the Si–O stretching region (1150–950 cm⁻¹).

## MMT- drug interactions

It has been noted that clay platelets reorganize as a result of clay particle disintegration caused by increased moisture levels. Current understanding suggests that controlled drug delivery with layered materials is achieved through the intercalation of drugs via ion exchange in inorganic silicates. These layered silicates form supramolecular assemblies with a lamellar structure, where the drug is embedded between the silicate layers. To promote intercalation, solid substrates, especially ion exchangers, can be combined with an ionic drug in solution [29]. “Counter-ions” can extract the drug from the substrate in body fluids and deliver it to the body. The exchanger can then be discarded or decomposed. Clay minerals, being naturally occurring inorganic cationic exchangers, can exchange ions with common pharmaceuticals in solution [30]. Smectites, especially MMT and saponite, are frequently studied because they possess higher cation exchange capacities than other therapeutic silicates, such as talc, kaolin, and fibrous clay minerals. The functional groups, the specific clay mineral involved, and the physical-chemical properties of the organic molecules all influence the suitability of a given process [31].

## Drug release from MMT and body absorption mechanisms

There are numerous ways for drugs and clay to interact or become complex thanks to the structure of smectite clays:

i. Drugs that are cationic and use cation exchange: This results in a relatively solid drug-clay connection.

for extending drug release, for instance, on the platelet faces.

Weak anion exchange is caused by anionic medicines at the margins of platelets.

iii. Platelet faces forming hydrogen bonds.

iv. Intercalation, which may also entail cation exchange between un-delaminated platelets.

v. Solvent adsorption to quicken the rate of dissolution of hydrophobic contacts or weakly soluble drugs on the vast surface area of the clay. Protonation, ligand exchange, pH-dependent charge sites, cation bridges, and water bridges are a few examples (van der Waals).

Clay particles were combined with aqueous drug solutions, left to reach equilibrium, and then the solid phases were retrieved and dried to produce the clay-drug mixture. Additionally:

(a) Nanoclay dispersions were induced to coagulate to entrap bioactive chemicals.

(b) A dry method was also described, involving either mixing the clay and the drug directly or bringing them into contact with each other at the drug’s melting point (particularly useful for weakly soluble compounds) [32].

### Factors affecting adsorption of drugs onto MMT

The extent to which pharmaceuticals are adsorbed onto MMT interlayers is affected by several factors. Kulshrestha et al. examined how pH influences the adsorption of oxytetracycline on native MMT, Na-MMT, and HDTMA MMT. They found that adsorption decreased with increasing pH, following the sequence: pH 1.5 > 5.0 > 8.7 > 11.0 for both native and sodium forms of MMT. While temperature can slightly impact drug adsorption, its effect is generally less significant compared to pH. For example, increased temperatures inhibited the adsorption of vitamin B6 on MMT, while, on the contrary, induced increased temperature lead to more adsorption of fluoride.

Another parameter in adsorption is ionic strength; in general, increasing ionic strength decreases the adsorption coefficient (Kd) although this does not hold true for all the drugs. The initial concentration also counts, as the drug concentration increases, and consequently higher amounts of drug are adsorbed on MMT, up to saturation, after which, more will not be retained. Like any other clay mineral, MMT has a cation exchange capacity (CEC), indicating number of exchangeable cations for each kilogram of material. Apart from being adsorbed, drugs can also deteriorate within the MMT interlayer.

## Toxicity

Previous research indicated that using MMT as a carrier for the anticancer drug 5-Fluorouracil (5-FU) is pharmaceutically viable. Consequently, this study aims to evaluate the toxicity of MMT to assess its clinical safety. In the study, hematological data from rats administered oral MMT showed significantly higher levels of hemoglobin (Hb), hematocrit, and red blood cell (RBC) counts compared to rats given oral PBS buffer (*p* < 0.05). However, no statistically significant differences were observed between the experimental and control groups regarding mean corpuscular volume (MCV), mean corpuscular hemoglobin (MCH), mean corpuscular hemoglobin concentration (MCHC), white blood cell (WBC) count, or WBC differential count (*p* > 0.05). Similarly, after intravenous injection, there were no significant differences in hematological parameters between the experimental and control groups (*p* > 0.05). In the bacterial model using Saccharomyces cerevisiae, a non-pathogenic yeast, various doses of MMT, MMT-K6Fe(CN)3 (MMTK), and MMT-K6Fe(CN)3-sodium alginate (MMTK-SA) were tested. Toxicity ranking in accordance with either EC0 or EC50 line graded Lannate as the most toxic agent, followed in order by MMTK, MMTK-SA, MMT-SA, and MMT itself. It appears that potassium hexacyanoferrate and Lannate are both significantly more toxic in comparison to MMT when taken alone. MMT, therefore, seems to be relatively safer when tested on S. cerevisiae as well as Wistar rats. Chronic toxicity tests in these models showed that MMT when looked at from a dose-response point of view was generally non-toxic to S. cerevisiae. Moreover, biostatistical analysis showed only minor deviations in toxicity between high-dose MMT and PBS, according to hematological, biochemical, and histopathological evaluations. Overall, MMT was mostly stated as non-toxic to Wistar rats.

**Fig. 2:**
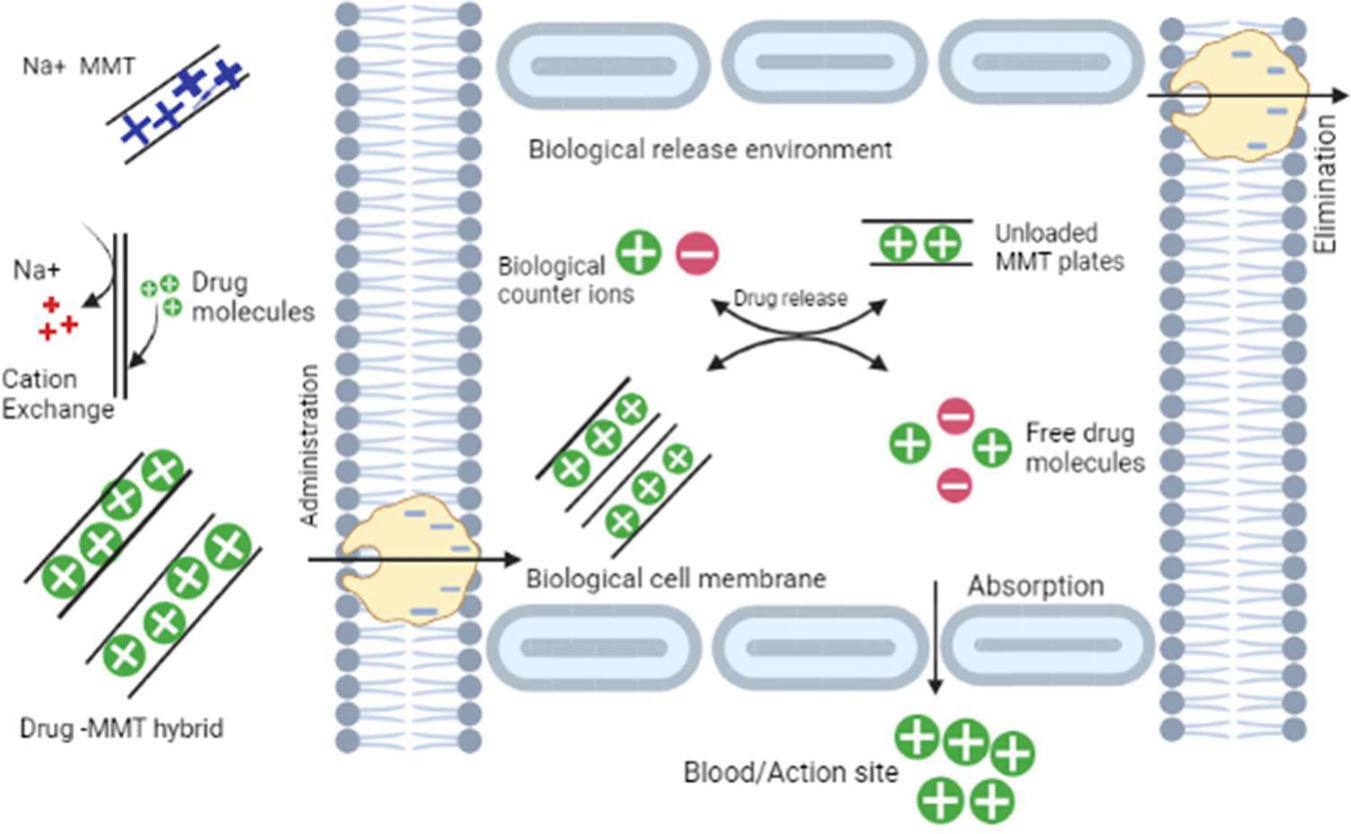
Drug release mechanism of MMT

Toxicity outcomes between pristine and functionalized montmorillonite (MMT) differ considerably and deserve distinct definitions. Generally, pristine MMT exhibits low acute toxicity; however, at higher doses or following long-term exposure because of the high surface reactivity and negative charge of the particles, it may induce nonspecific protein adsorption, membrane irritation, aggregation, and inflammatory responses. Functionalized MMT is generally polymer coated, PEGylated, or chitosan-modified and usually has a higher biocompatibility profile, is less prone to aggregation, has better colloidal stability, and permits controlled drug release, thus resulting in reduced cellular and systemic toxicity. This is a very acknowledged distinction because toxicity of pristine MMT cannot be directly extrapolated to pharmaceutically relevant modified systems, which lie at the essence of clinical-oriented formulations [Fig. 2].

## Scientific prospection

The continuous increase in research on various methods and techniques to impart greater safety, efficacy, and patient compliance to drug delivery systems is a welcome development. Clay minerals, sometimes even acting as active ingredients, can often be found as excipients in pharmaceutical formulations. While it is well studied how various drugs might interact with clays while given simultaneously, the exploration of how these clays may modify drug release is fairly recent. Clays, in the recent past, have been acknowledged for their ability to regulate drug delivery owing to high retention capacities, swelling properties, and colloidal behavior.

**Fig. 3:**
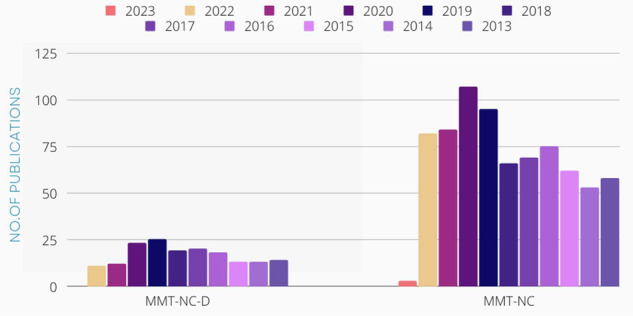
Publications trends for MMT-NC and MMT-NC-D

A review of the PubMed database from 2013 to 2023 with the searching terms “MMT nanocomposites drug” and “Montmorillonite nanocomposites” [Title/Abstract] of 661 publications on MMT nanocomposites. In these decades, different MMT nanocomposite drugs have been developed with findings presented in 139 papers. Before 2013, 58 journals listed articles on MMT-NC, but after 2014, the number began to rise sharply, peaking at 107 articles in 2020. In 2022, 82 manuscripts were published, and by the end of 2023, this number might go close to 100. On the contrary, research on MMT-NC-D(Montmorillonite-Nanocomposite-Drug) (repurposed) accounted for only 139 manuscripts from 2013 to 2023, with two clear publication peaks in 2019 and 2020, likely propelled by the ongoing SARS-CoV-2 pandemic. The increasing publication output on MMT has highlighted great relevance and applications in various areas, including biotechnology, pharmaceuticals, material science, microbiology, and immunology, showcasing its rising significance [Fig. 3].

Mucoadhesive bio-nanocomposite hydrogels have been developed with the intent of prolonging the residency time of drugs in the stomach. These bio-nanocomposite hydrogels employ tripolyphosphate as a cross-linking agent, chitosan in the form of a bioadhesive matrix, and MMT to modulate drug release. MMT-famotidine and chitosan bio-nanocomposite hydrogels demonstrated reputable sustained drug release characteristics together with acceptable mucoadhesion and prolonged retention in the stomach. From the study, it can be concluded that these mucoadhesive bio-nanocomposite hydrogels might improve therapeutic efficacy and bioavailability of famotidine when administered via the oral route even to a greater extent [33].

They have been widely used in cancer therapy; however, doxorubicin (DOX) faces rapid clearance from the body, poor permeability, and low solubility. To combat these problems, a pH-responsive nanocomposite was synthesized using chitosan (CS), MMT, and nitrogen-doped carbon quantum dots (NCQDs). The system was loaded with DOX using a double emulsion system for prolonged sustained release. The CS-MMT-NCQD hydrogel exhibited enhanced load and entrapment efficiency for DOX. In addition, the presence of NCQDs resulted in a prolonged pH-responsive release of DOX over 96 hours, compared to CS-MMT-DOX nanocarriers at pH 5.4. From the MTT cytotoxicity assay, it was revealed that the DOX-loaded CS-MMT-NCQDs hydrogel was significantly more cytotoxic toward MCF-7 than free DOX or CS-MMT-NCQDs (*p* < 0.001). The presence of NCQD nanoparticles also enhanced apoptosis induction, as evidenced by flow cytometry. Therefore, the results suggested that DOX-loaded MMT nanocomposite is a promising candidate for selective induction of apoptosis in cancer cells.

Shabnam Haseli and others, in yet another work, worked on various parameters like loading efficacy, release sustainability, and anti-cancer efficiency of curcumin. A curcumin-loaded nanocomposite hydrogel system was designed with MMT nanoparticles in combination with a chitosan (CS) and agarose (Aga) hydrogel (Table 4). The CS-Aga-MMT-Cur nanocomposite induced cytotoxicity in MCF-7 cells, comparable to curcumin alone (*p* < 0.001). The improved release from the nano-emulsion and inclusion of MMT to the hydrogel led to an increase in the percentage of apoptotic cells. This delivery system enhanced curcumin loading and sustained release and improved its anti-cancer activity, thereby holding prospects for overcoming the restrictions faced by curcumin in cancer treatment applications [34].

**Table 4:** Summary of MMT application as drug carrier

S. No	Clay material	Drug	Polymer	Route of delivery	Reference
1	MMT	Clozapine Curcumin Carbamazipine	PVP HPMC SDS	Oral	[39]
2	MMT	Trimethoprim sulfamethoxazole	Tin oxide	Topical	[40]
3	MMT	–	Chitosan PVA	Topical	[41]
4	MMT	Chloramphenicol	Agar Carrageenan	Topical	[35]
5	MMT	Metronidazole	Cetyl trimethyl Ammonium bromide	Topical	[42]
6	MMT	Atenolol Acebutalol	Ammonium rhodonide Citric acid	Oral	[43]
7	MMT	Ciproflaxacin	Gelatin	Topical	[44]
8	MMT	Neomycin	Carboxymethyl cellulose	Topical	[45]
9	MMT and Halloysite	Chondroitin Sulfate	Chitosan	Topical	[46]
10	MMT	Ammonium persulphate	Sodium carboxymethyl cellulose	Topical	[14]

Agar/carrageenan/MMT hydrogels were developed to assess their compatibility as wound dressing materials and to explore the effects of the MMT content on other properties of the materials. The hydrogel compositions containing CLP(Chloramphenicol) demonstrated efficient antibacterial activity against S. aureus and E. coli. Toxicological studies showed that MG-63 cells tolerated the hydrogels well. Agar/carrageenan hydrogels put up with LDC(Lidocaine) and CLP and agar/carrageenan/MMT hydrogels containing 5% MMT, showing ultimate compressive stresses of 38.30 kPa and 47.70 kPa, respectively. In view of the experimental results, agar-carrageenan and agar-carrageenan MMT hydrogels show great promise for applications in wound care [35]. Yet, MMT shown extensive application as a drug carrier for various drugs and the results were summarized in Table 4.

## Technological prospection

**Fig. 4:**
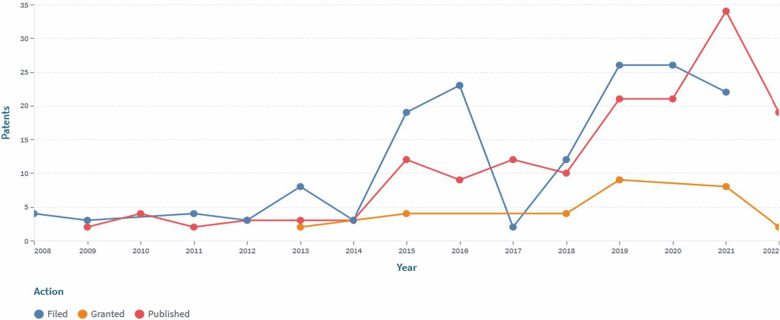
Trend of patents filed, published and granted from 2008 to 2022

Following the retrieval of clinical data, the search for MN patents continued. Patent literature was explored through various databases, including the USPTO (United States Patent and Trademark Office), EPO (European Patent Office), WIPO (World Intellectual Property Organization), and others. The search used the query (Montmorillonite AND (nanocomposite AND (drug AND delivery))). No filters were applied to the search. A total of 482 patent filings across different jurisdictions were identified. Figure 4 illustrates the number of patents filed, published, and granted from 2008 to 2022. Table 5 provides a summary of notable patents released in 2021 and 2022. A significant surge in published patents was observed in 2017, highlighting the adoption of MMT in advanced technologies aimed at enhancing therapeutic efficacy. The pattern of patent filings exhibited fluctuations, while the number of issued patents showed a notable increase, a trend that is anticipated to continue into 2023 (Fig. 4).

**Table 5:** Summary of latest patents on MMT-NC

Jurisdiction	Publication year	Application number	Application date	Title	Applicants
US	2022	US 202117483022A	23-09-2021	Gels derived from poly(ethylidene norbornene)-b-poly(cyclopentene) block copolymer nanocomposites for viscosity modifications and drilling fluid applications	Univ Iowa State Res Found Inc
US	2022	US 201917415987A	18-12-2019	Nanomaterials, nanocomposite materials, and methods thereof	Uti Lp
US	2022	US 201816493459A	13-03-2018	Nanocomposite ionic-covalent entanglement reinforcement mechanism and hydrogel	Texas A & M Univ Sys
US	2022	US 202017627800A	14-07-2020	Rigid resorbable materials with polymer and organic fillers	Evonik Operations Gmbh
US	2022	US 202117527790A	16-11-2021	Methods and systems of three dimensional printing	Univ Florida
WO	2022	KR 2021003974W	31-03-2021	Methods for encapsulation of spermatozoa and cryopreservation of encapsulated spermatozoa	Noah Biotech Inc
US	2022	US 201916594454A	07-10-2019	Iron oxide modified halloysite nanomaterial	Qatar Found Education Science & Community Dev
US	2022	US 202117497495A	08-10-2021	Poly(ethylene terephthalate)-graphene nanocomposites from improved dispersion	Niagara Bottling Llc
EP	2022	EP 18723613A	18-04-2018	Biobased super-absorbing polymers	Univ Delft Tech
US	2022	US 201916371235A	01-04-2019	Methods for adhering tissue surfaces and materials and biomedical uses thereof	Inserm Institut De La Sante Et De La Rech Medicale; Group
WO	2022	US 2021/0055434W	18-10-2021	Compositions for inducing tumor immunity and reducing drug tolerance	Brigham & Womens Hospital Inc
US	2022	US 202017066174A	08-10-2020	Boron nitride polymer composite foam derived from emulsions stabilized by boron nitride kinetic trapping	Univ Connecticut
US	2022	US 202017597680A	16-07-2020	Improved homology dependent repair genome editing	Inari Agriculture Inc
US	2022	US 202117338274A	03-06-2021	Method and apparatus for treating bone fractures, and/or for fortifying and/or augmenting bone, including the provision and use of composite implants, and novel composite structures which may be used for medical and non-medical applications	206 Ortho Inc
US	2022	US 201716336276A	28-09-2017	Nanofiber structures and methods of use thereof	Univ Nebraska
US	2022	US 201816647690A	19-09-2018	Methods for producing a nanofiber or microfiber structure	Univ Nebraska
US	2022	US 201113014632A	26-01-2011	Stent and stent delivery system with improved deliverability	Mcclain James B;;Taylor Douglas;;Enscore David;;Mt Acquisition Holdings Llc
US	2021	US 201916560394A	04-09-2019	Gels derived from poly(ethylidene norbornene)-b-poly(cyclopentene) block copolymer nanocomposites for viscosity modifications and drilling fluid applications	Univ Iowa State Res Found Inc
US	2021	US 201916518626A	22-07-2019	Facile clay exfoliation using polymer silicone surfactants	Industrial Science & Tech Network Inc
US	2021	US 201616060273A	21-12-2016	Mineral-based nanoparticles for arthritis treatment	Texas A & M Univ Sys
US	2021	US 202117332936A	27-05-2021	System and method for treating medical sewage containing sars-cov-2 based on nano graphene	Univ Shaoguan;;Foshan Qionglu Health Tech Co Ltd
US	2021	US 201916517124A	19-07-2019	Rigid resorbable materials with polymer and organic fillers	Evonik Operations Gmbh
WO	2021	EP 2020069864W	14-07-2020	Rigid resorbable materials with polymer and organic fillers	Evonik Operations Gmbh
US	2021	US 201716334202A	18-09-2017	Methods and systems of three dimensional printing	Univ Florida
US	2021	US 201816231291A	21-12-2018	Mechanically robust aerogels and preparation method thereof	Pashaei Soorbaghi Fatemeh
US	2021	US 201715635159A	27-06-2017	Spray-coating method with particle alignment control	Massachusetts Inst Technology;;Univ Khalifa Science & Technology
US	2021	US 202117155748A	22-01-2021	Anti-inflammatory, disinfecting and accelerated healing gel for wounds and lesions	Nanocare Pharma Inc

**Fig. 5:**
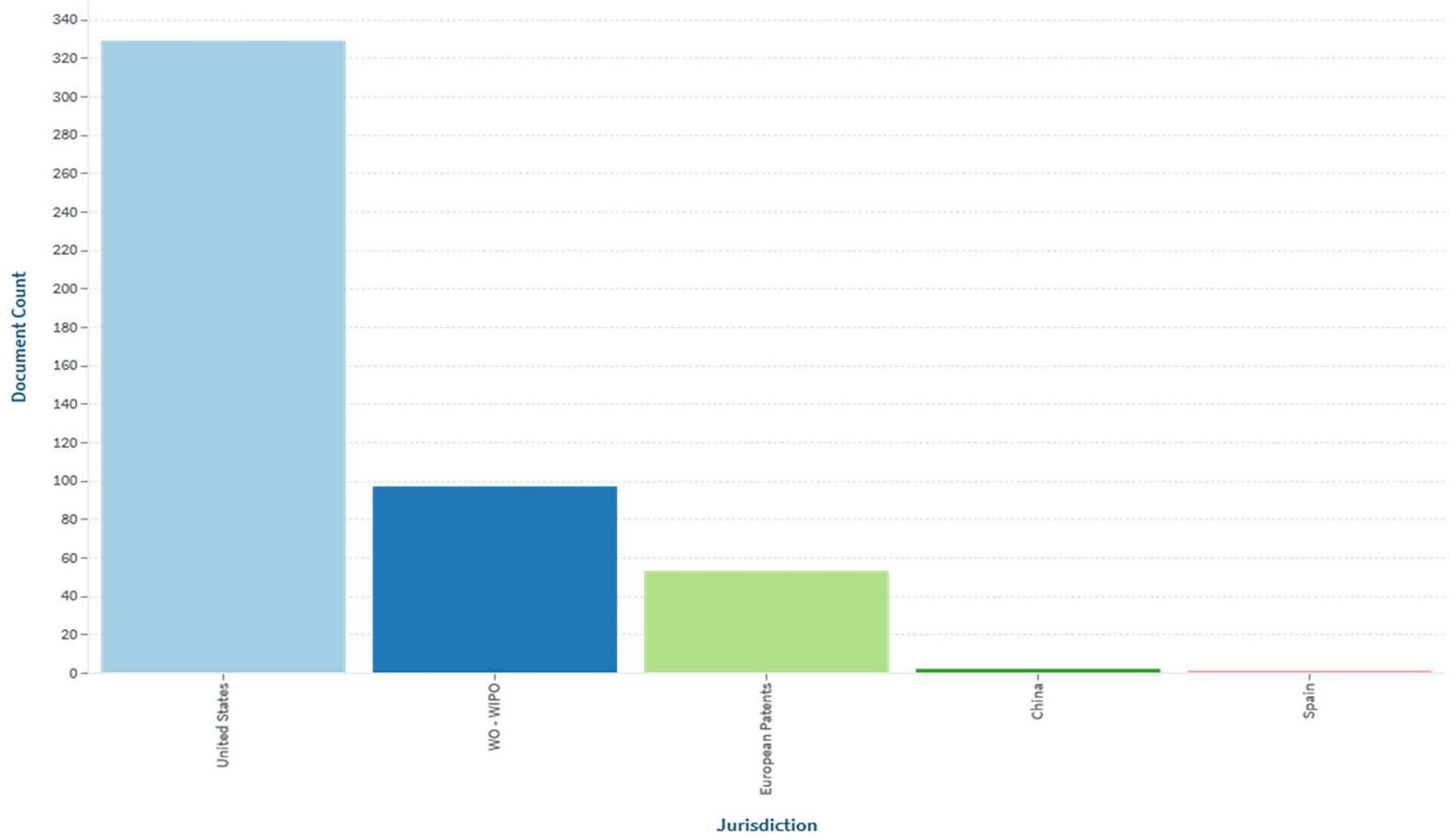
Patents documents by jurisdiction

Figure 5 illustrates the extensive distribution of patents across various jurisdictions. Leading the count with 329 documents is the United States, followed by WO-WIPO with 97 patents. European patents described 53 inventions, with 2 filings under Chinese law. Additionally, patent applications were filed in several other countries, including Australia, the Republic of Korea, Japan, and Russia, among others.

**Fig. 6:**
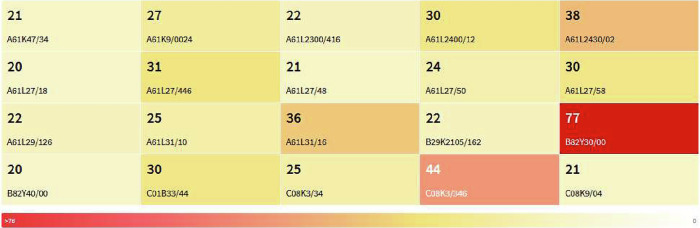
Top CPC classification codes with heat map

The grouping of filed patents based on their technical qualities is made easier by patent classification. Additionally, it facilitates the most precise and accurate patent search. Because of their patent rules, various governments utilize a variety of classifications. All jurisdictions stepped forward to create standard technical codes to combine the patents. Cooperative patent categorization (CPC) and international patent classification are the two categories that they fall under (IPC). From Q4 of 2010, CPC entered the picture as an addition to IPC. USPTO and EPO handled all management of CPC [36, 37]. MMT patents were categorized, and Fig. 6 shows the schematic distribution (Fig. 6).

The B82Y class encompasses patents related to specific uses or applications of nanostructures, including their measurement, analysis, manufacture, or treatment. Within this class, subclass B82Y30/00 focuses on nanotechnology for materials or surface science, such as nanocomposites. This subclass includes 77 patents, highlighting the relevance of MMT in nanocomposites. The C08K class covers the use of inorganic or non-macromolecular organic substances as ingredients in paints, inks, varnishes, dyes, polishes, and adhesives. Notably, C08K3 ranks second with 44 patents specifically addressing inorganic substances used in these applications. Another significant class, A61L, contains a substantial number of patents related to various methods and apparatuses for sterilizing materials, disinfection, and the chemical aspects of medical supplies. This class includes inventions related to sterilization, preservation of food, and medical preparations. For instance, subclass A61L2430/02 is concerned with materials for bone reconstruction and weight-bearing implants, while other subclasses such as A61F2/07 and A61F2/82 focus on materials for surgical articles, stents, and surgical gloves. These patents are protected under A61L31/00, indicating a wide range of applications for materials used in medical and surgical contexts [38].

**Fig. 7:**
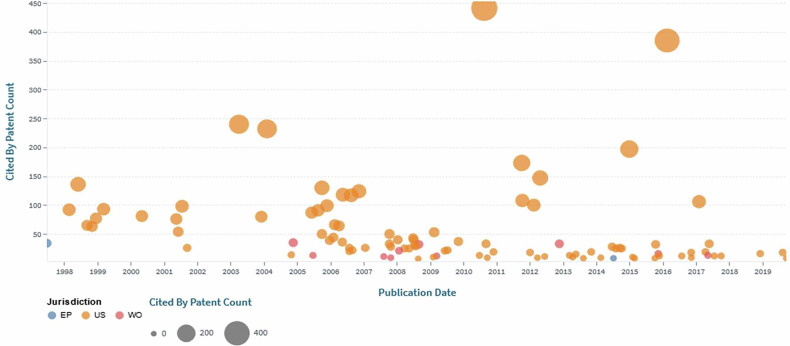
Patents cited by publication date

The number of citations was also taken into account as a metric of patent suitability. The filing date and the quantity of patients with citations for various jurisdictions are shown in Fig. 7. The relevance was determined to be greater than 100 and the cited patent counts were higher for the patents produced in 2005–2014. Many of the USPTO-published patents displayed the greatest number of citations within these specified times.

### Technical aspects of few patents on MMT

Patent WO 2014/021800 A2 outlines a green technology approach focused on the processing of micro- and nano-sized clay particles derived from mineral resources in Turkey. The method involves utilizing novel functional organic intercalants to prepare, purify, and modify clay particles, particularly bentonite and MMT clays, for the creation of advanced polymer-layered silicate nanomaterials.

The technology emphasizes the production of a new generation of intercalated organic derivatives and exfoliated polymer-layered silicate nanocomposites, including nanofilms, nanocoatings, nanofibers, and nanomedical materials. The procedure is composed of different stages such as double granulation-milling of raw mineral material, microwave-sonication of particle preparation, and press filtering or ultracentrifuging for isolation of the water/ethanol mixture. In addition, membrane separation or distillation columns are also used in order to evaporate ethanol from water/ethanol mixtures, followed by recycling isolated ethanol, water, and saltwater with pH control. The final remains of the process involve double cyclonation for the preparation of particles along with their selection, which yields particles smaller than 240–270 nm having a very high Zeta potential of 72–103 mV. This approach in clay particle processing promises advances in high-performance engineering and bioengineering polymer-layered silicate nanomaterials, which can be used to revolutionize several sectors and applications.

The kit and bioadhesive sealant is very unique and patented in the US (US 2019/0083676 A1) as it combines gelatin, alginate, MMT, and a coupling agent in a composite bio-adhesive sealant. Also, this adhesive exhibits rapid curing, optimum viscosity, high burst strength, flexibility, biocompatibility, and biodegradability, making it suitable for numerous medical applications.

Moreover, a self-assembled nanocomposite can be produced using a smectic clay and an oligosilsesquioxane adduct such as solvent assistance in self-assembly and formation of microcapsules. The method of making such microcapsules will be dissolving a nanocomposite in a fit volatile solvent and allowing it to evaporate the solvent. In essence, a guest-encapsulated micro capsule can be made by dissolving into a fit volatile solvent the nanocomposite and guest compound and evaporating.

**Fig. 8:**
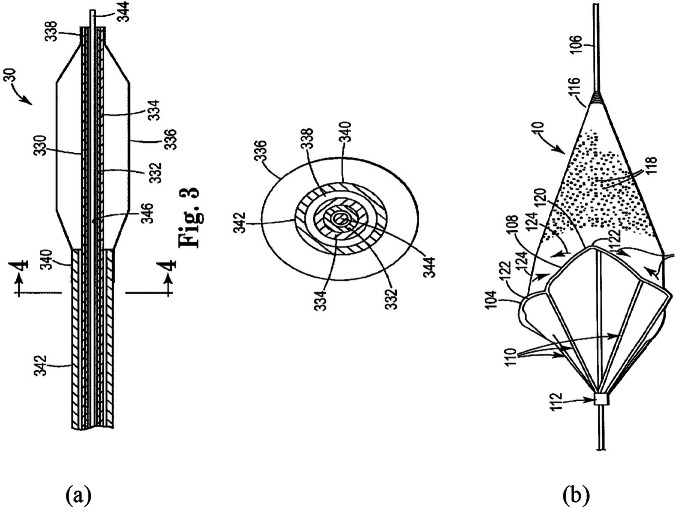
**a** MMT nanocomposite in making the medical devices and **b** Cardiovascular balloon catheter containing MMT-NC

Also, MMT nanocomposites are patented for use in therapeutic procedures and medical devices in US patent US 2003/0065355 A1. The achievement of nanoparticles by MMT will open the door for different capabilities and advantages to be incorporated into such medical devices. Instructions for fabricating such newly developed materials, which are anticipated to impart certain advantages to their users, are contained in the patent. As such, these inventive devices are likely to devise new methods of medical care-treatment or diagnostics-acquired through their novel features and functionalities (Fig. 8).

Many inventive ideas have been brought to the limelight in clay-based materials over different disciplines in which their applicability and beneficial use have been divergent:

EP 2319453 B1 describes cardiovascular balloon construction using nanocomposites (NC). The invention indicates the specific physical properties of nanoparticles and nanocomposites, which could be used in the sector of medical devices and might open up ideas for the development of innovative solutions in the field of medical device manufacturing.

US 2012/0289618 A1 presents a method for clay/polymer composites with improved thermal and mechanical stabilization employing a supercritical fluid-organic solvent system to produce ecofriendly and simple, cost-effective clay/biodegradable polymer stereoisomeric nanocomposites with a bright promise for several applications, including use in medical devices.

US 2019/0255221 A1 describes the application of MMT and clay materials in the form of scaffolds that could be used for bone regeneration. Combinations of biocompatible polymers and clay constitute these compositions proven to function as scaffolds in bone repair, particularly for major bone defects. Furthermore, it can combine different scaffolds to form scaffold blocks to increase versatility and adaptability in bone repair applications.

Univ King Fahd Pet & Minerals patented a method to lessen the proliferation or viability of melanoma cells by applying nano clay. In addition, it covers pharmacological formulations for treatment of melanoma that incorporate nanoclay. This concept shows the potential that nanoclay could harness in establishing new therapeutic developments for melanoma and potentially other diseases.

This demonstrates how clay materials are quite different and applicable in various fields-from medical devices to bone regeneration and finally cancer treatment. Their properties are unique, making them essential components of novel remedies tailored towards improving human health and well-being. MMT has proven versatility by being one of the outstanding ingredients in the development of vaccines. An example is an oral vaccine (containing pathogens in a non-infectious form) combined with a layered silicate, which attaches to, encapsulates, and thereby protects the pathogen. Patent WO 2015/030613 A1 covers the use of layered silicates in vaccines, giving preference to materials such as MMT, bentonite, kaolinite, or combinations of these.

Furthermore, the invention disclosed in CN 104666250A describes a method of preparing chlortetracycline sustained-release microspheres in which chlortetracycline is mixed with MMT and added to water to form a suspension, which is then added to a sodium alginate solution. After having properly mixed, the resulting mixture is dropped into a calcium chloride solution for crosslinking. The gel spheres obtained are collected and air-dried to produce chlortetracycline sustained-release microspheres.

Such illustrative examples demonstrate the various applications of MMT in pharmaceuticals-from vaccine formulation to drug delivery systems-revealing its favorability in enhancing therapeutic outcome and patient care.

## Limitations of MMT-based drug delivery

While montmorillonite (MMT)-filled polymer nanocomposites have shown great promise in pharmaceutical nano drug delivery, numerous limitations have been reported in available literature. MMT being of natural origin causes variability in the fine mineral composition, cation-exchange capacity, and the level of impurities on batch-to-batch basis, which can, in turn, affect drug loading, swelling behavior, and release reproducibility. In some cases, weak electrostatic drug interactions with MMT layers cause burst or early drug release in the physiological environment, thus impairing sustained delivery. Attaining uniform dispersion and stable exfoliation of MMT within the polymer matrices is still a challenge, particularly at high clay loadings, which could lead to aggregation and inconsistent performance. The availability of long-term biodegradability, biodistribution, and chronic toxicity data remains limited with most studies focusing on short-term in vitro evaluations. Moreover, the escalating need for clinical translation proposes that issues of scalability, sterilization, and regulatory compliance are rarely given sufficient attention in academic reports, as noted in patent literature.

## Future prospection and conclusion

This review elucidates certain applications of polymer nanocomposites filled with montmorillonite (MMT) for pharmaceutical nano drug delivery systems, constituting a very important class of materials. They are tunable layered materials, having very high surface area, ion-exchange capacity, and modulated drug-loading and release characteristics. According to the survey of the literature, polymer-MMT nanocomposites were better than other existing formulation systems in improving stability against drug degradation, dissolution, enhanced bioavailability, and control release profiles. Patent analysis further highlights that these systems have clearly been placed in formulation optimization, surface modifications, and large-scale processing methodologies to underline their translational relevance concerning industrial and clinical applications. The joint scholarly and patent appraisal identifies crucial structure-property-performance relationships, particularly the manipulation of exfoliation and intercalation of nanostructures for enhanced therapeutic efficacy and formulation robustness. Patent trends suggest a notable consideration toward designing multifunctional nanocomposites with enhanced biocompatibility and manufacturability without regulatory considerations-an often overlooked aspect in academic endeavors. This denotes the existence of gaps between laboratory scale innovations and the product-oriented approach. Future research should be oriented toward formulation design with Quality-by-Design (QbD), mechanistic insight into polymer-clay-drug interactions at the molecular level, and systematic evaluation of performance in vivo and long-term safety profile. Incorporating Quality by Design (QbD) into upcoming MMT formulations will involve a sequential approach starting from an inquisitive Quality Target Product Profile (QTPP) and coring it into Critical Quality Attributes (CQAs), such as particle size, degree of exfoliation, drug loading, release kinetics, and biocompatibility. Use risk assessment tools, such as Ishikawa diagrams and FMEA, to identify Critical Material Attributes (CMAs) of MMT, including source, purity, cation type, and surface modification, and Critical Process Parameters (CPPs)—mixing method, clay concentration, pH, and drying method. Design of Experiments (DoE) can subsequently be applied to optimize formulations within a defined design space. Scalable manufacturing would include using melt compounding, high-shear mixing, spray drying, or continuous manufacturing, which are all compatible with industry processes, yet could guarantee reproducibility, sterility, and GMP compliance. Early integration of scale-up considerations, in-line process analytical technologies (PAT), and robust quality control would make the clinical translation of an MMT-based delivery system smoother.

A more concerted effort between academic research and patented processes in the areas of scalability, reproducibility, and regulatory affairs would greatly aid clinical translation. Ultimately, this integrated scholarly-patented view strengthens the argument of montmorillonite-filled polymer nanocomposites as highly promising but complex platforms for the future generation of pharmaceutical nano-drug delivery systems while giving a clear direction for future developments and commercialization.

## References

[1] Balderson BH, Grothaus L, Harrison RG, McCoy K, Mahoney C, Catz S. Chronic illness burden and quality of life in an aging HIV population. AIDS Care. 2013;25:451–8. 10.1080/09540121.2012.712669. 22894702 10.1080/09540121.2012.712669 PMC3535557

[2] Omanović-Mikličanin E, Badnjević A, Kazlagić A, Hajlovac M. Nanocomposites: a brief review. Health Technol. 2020;10. 10.1007/s12553-019-00380-x.

[3] Hoque ME, Ramar K, Sharif A. Advanced Polymer Nanocomposites: Science, Technology and Applications, 2022. 10.1016/C2020-0-01039-X.

[4] Thostenson E, Li C, Chou T. Nanocomposites in context. Compos Sci Technol. 2005;65:491–516. 10.1016/j.compscitech.2004.11.003.

[5] Bergna HE, Roberts WO (Eds.). Colloidal Silica: Fundamentals and Applications (1st ed.). CRC Press. 2005. 10.1201/9781420028706.

[6] Armstrong G. An introduction to polymer nanocomposites. Eur J Phys. 2015;36:063001. 10.1088/0143-0807/36/6/063001.

[7] Bórquez-Mendivil A, Hurtado-Macías A, Leal-Pérez JE, Flores-Valenzuela J, Vargas-Ortíz RÁ, Cabrera-Covarrubias FG, et al. Hybrid coatings of SiO_2_–recycled PET unsaturated polyester resin by sol-gel process. Polymers. 2022;14:3280. 10.3390/polym14163280. 36015537 10.3390/polym14163280 PMC9415624

[8] Moraes JDD, Bertolino SRA, Cuffini SL, Ducart DF, Bretzke PE, Leonardi GR. Clay minerals: properties and applications to dermocosmetic products and perspectives of natural raw materials for therapeutic purposes—a review. Int J Pharm. 2017;534:213–9. 10.1016/j.ijpharm.2017.10.031. 29038067 10.1016/j.ijpharm.2017.10.031

[9] Bergaya F, Detellier C, Lambert JF, Lagaly G. Introduction to clay-polymer nanocomposites (CPN). Dev Clay Sci. 2013. 10.1016/B978-0-08-098258-8.00020-1.

[10] Konta J. Clay and man: clay raw materials in the service of man. Appl Clay Sci. 1995;10:275–335. 10.1016/0169-1317(95)00029-4.

[11] Rieder M, Cavazzini G, D’Yakonov YS, Frank-Kamenetskii VA, Gottardi G, Guggenheim S, et al. Nomenclature of the micas. Can Mineral. 1998;36. 10.1180/minmag.1999.063.2.13.

[12] Mishra N, Wani TU, Rashid M, Kumar M, Chaudhary S, Kumar P. Targeting aspects of nanogels: an overview. Int J Pharm Sci Nanotechnol. 2014;7. 10.37285/ijpsn.2014.7.4.3.

[13] Thakur G, Singh A, Singh I. Chitosan-montmorillonite polymer composites: formulation and evaluation of sustained release tablets of aceclofenac. Sci Pharm. 84. 10.3390/scipharm84040603. 10.3390/scipharm84040603 PMC5198020 28656939

[14] Sharifzadeh G, Hezaveh H, Muhamad II, Hashim S, Khairuddin N. Montmorillonite-based polyacrylamide hydrogel rings for controlled vaginal drug delivery. Mater Sci Eng C. 2020;110:110609. 10.1016/j.msec.2019.110609. 10.1016/j.msec.2019.110609 32204060

[15] Sun W, Wei Z, Sun D, Liu S, Fatahi B, Wang X. Evaluation of the swelling characteristics of bentonite-sand mixtures. Eng Geol. 2015;199:1–11. 10.1016/j.enggeo.2015.10.004.

[16] Subramanian T, Dhakshinamoorthy A, Pitchumani K. Amino acid intercalated layered double hydroxide catalyzed chemoselective methylation of phenols and thiophenols with dimethyl carbonate. Tetrahedron Lett. 2013;54:7167–70. 10.1016/j.tetlet.2013.10.098.

[17] Komine H. Simplified evaluation for swelling characteristics of bentonites. Eng Geol. 2004;71. 10.1016/S0013-7952(03)00140-6.

[18] Fudala Á, Pálinkó I, Kiricsi I. Preparation and characterization of hybrid organic-inorganic composite materials using the amphoteric property of amino acids: amino acid intercalated layered double hydroxide and montmorillonite. Inorg Chem. 1999;38:4653–8. 10.1021/ic981176t. 11671187 10.1021/ic981176t

[19] Fernandez R, Martirena F, Scrivener KL. The origin of the pozzolanic activity of calcined clay minerals: a comparison between kaolinite, illite and montmorillonite. Cem Concr Res. 2011;41:113–22. 10.1016/j.cemconres.2010.09.013.

[20] Plaza De Los Reyes C, Vidal G. Effect of variations in the nitrogen loading rate and seasonality on the operation of a free water surface constructed wetland for treatment of swine wastewater. J Environ Sci Heal Part A Toxic. 2015;50. 10.1080/10934529.2015.1059106. 10.1080/10934529.2015.1059106 26252764

[21] Glinskikh VN, Nesterova GV, Epov MI. Forward modeling and inversion of induction logs from shaly sand reservoirs using petrophysical conductivity models. Russ Geol Geophys. 2014;55:793–9. 10.1016/j.rgg.2014.05.022.

[22] Idumah CI. Thermal expansivity of polymer nanocomposites and applications. Polym Technol Mater. 2023;62. 10.1080/25740881.2023.2204952.

[23] Anderson RL, Ratcliffe I, Greenwell HC, Williams PA, Cliffe S, Coveney PV. Clay swelling - a challenge in the oilfield. Earth Sci Rev. 2010;98:201–16. 10.1016/j.earscirev.2009.11.003.

[24] Nomicisio C, Ruggeri M, Bianchi E, Vigani B, Valentino C, Aguzzi C, et al. Natural and synthetic clay minerals in the pharmaceutical and biomedical fields. Pharmaceutics. 2023;15:1368. 10.3390/pharmaceutics15051368. 37242610 10.3390/pharmaceutics15051368 PMC10220772

[25] Undabeytia T, Shuali U, Nir S, Rubin B. Applications of chemically modified clay minerals and clays to water purification and slow release formulations of herbicides. Minerals. 11. 10.3390/min11010009.

[26] Yin H, Song CQ, Suresh S, Wu Q, Walsh S, Rhym LH, et al. structure-guided chemical modification of guide RNA enables potent non-viral in vivo genome editing. Nat Biotechnol. 2017;35:1179–87. 10.1038/nbt.4005. 29131148 10.1038/nbt.4005 PMC5901668

[27] Uddin F. Montmorillonite: an introduction to properties and utilization. Curr. Top. Util. Clay Ind. Med. Appl. 2018. 10.5772/intechopen.77987.

[28] Hensen EJM, Smit B. Why clays swell. J Phys Chem B. 2002;106:12664–7. 10.1021/jp0264883.

[29] Browne JE, Feldkamp JR, White JL, Hem SL. Characterization and adsorptive properties of pharmaceutical grade clays. J Pharm Sci. 1980;69:816–23. 10.1002/jps.2600690719. 7391947 10.1002/jps.2600690719

[30] Lagaly G. Pesticide-clay interactions and formulations. Appl Clay Sci. 2001;18:205–9. 10.1016/S0169-1317(01)00043-6.

[31] Tolls J. Sorption of veterinary pharmaceuticals in soils: a review. Environ Sci Technol. 2001;35:3397–406. 10.1021/es0003021. 11563639 10.1021/es0003021

[32] Patel HA, Somani RS, Bajaj HC, Jasra RV. Nanoclays for polymer nanocomposites, paints, inks, greases and cosmetics formulations, drug delivery vehicle and waste water treatment. Bull Mater Sci. 2006;29:133–45. 10.1007/BF02704606.

[33] Farhadnejad H, Mortazavi SA, Jamshidfar S, Rakhshani A, Motasadizadeh H, Fatahi Y, et al. Montmorillonite-famotidine/chitosan bio-nanocomposite hydrogels as a mucoadhesive/gastroretentive drug delivery system. Iran J Pharm Res IJPR. 2022;21:127035. 10.5812/IJPR-127035. 10.5812/ijpr-127035 PMC9420228 36060919

[34] Haseli S, Pourmadadi M, Samadi A, Yazdian F, Abdouss M, Rashedi H, et al. A novel pH-responsive nanoniosomal emulsion for sustained release of curcumin from a chitosan-based nanocarrier: emphasis on the concurrent improvement of loading, sustained release, and apoptosis induction. Biotechnol Prog. 2022;38. 10.1002/BTPR.3280. 10.1002/btpr.3280 35678755

[35] Polat TG, Duman O, Tunç S. Agar/κ-carrageenan/montmorillonite nanocomposite hydrogels for wound dressing applications. Int J Biol Macromol. 2020;164:4591–602. 10.1016/j.ijbiomac.2020.09.048. 32931832 10.1016/j.ijbiomac.2020.09.048

[36] Kurakula M. Prospection of recent chitosan biomedical trends: evidence from patent analysis (2009–20). Int J Biol Macromol. 2020;165:1924–38. 10.1016/j.ijbiomac.2020.10.043. 33068625 10.1016/j.ijbiomac.2020.10.043

[37] Islam MM, Naveen NR, Anitha P, Goudanavar PS, Rao GSNK, Fattepur S, et al. The race to replace PDE5i: recent advances and interventions to treat or manage erectile dysfunction: evidence from patent landscape (2016–2021). J Clin Med. 2022;11:3140. 35683526 10.3390/jcm11113140 PMC9181403

[38] Rao GSNK, Gowthami B, Naveen NR, Samudrala PK. An updated review on potential therapeutic drug candidates, vaccines and an insight on patents filed for COVID-19. Curr Res Pharmacol Drug Discov. 2021;2:100063. 10.1016/J.CRPHAR.2021.100063. 34870158 10.1016/j.crphar.2021.100063 PMC8498785

[39] Kumar A, Hodnett BK, Hudson S, Davern P. Modification of the zeta potential of montmorillonite to achieve high active pharmaceutical ingredient nanoparticle loading and stabilization with optimum dissolution properties. Colloids Surf B Biointerfaces. 2020;193:111120. 10.1016/j.colsurfb.2020.111120. 32505995 10.1016/j.colsurfb.2020.111120

[40] Vidal CB, dos Santos AB, do Nascimento RF, Bandosz TJ. Reactive adsorption of pharmaceuticals on tin oxide pillared montmorillonite: Effect of visible light exposure. Chem Eng J. 2015;259:865–75. 10.1016/j.cej.2014.07.079.

[41] Noori S, Kokabi M, Hassan ZM. Nanoclay enhanced the mechanical properties of poly(vinyl alcohol) /chitosan /montmorillonite nanocomposite hydrogel as wound dressing. Proc Mater Sci. 2015;11:152–6. 10.1016/j.mspro.2015.11.023.

[42] Khataee A, Kıranşan M, Karaca S, Sheydaei M. Photocatalytic ozonation of metronidazole by synthesized zinc oxide nanoparticles immobilized on montmorillonite. J Taiwan Inst Chem Eng. 2017;74:196–204. 10.1016/j.jtice.2017.02.014.

[43] Shi W, Chu Y, Xia M, Wang F, Fu C. The adsorption performance and micro-mechanism of MoS_2_/montmorillonite composite to atenolol and acebutolol: adsorption experiments and a novel visual study of interaction. Ecotoxicol Environ Saf. 2021;213:111993. 10.1016/j.ecoenv.2021.111993. 33578102 10.1016/j.ecoenv.2021.111993

[44] Kevadiya BD, Rajkumar S, Bajaj HC, Chettiar SS, Gosai K, Brahmbhatt H, et al. Biodegradable gelatin-ciprofloxacin-montmorillonite composite hydrogels for controlled drug release and wound dressing application. Colloids Surf B Biointerfaces. 2014;122:175–83. 10.1016/j.colsurfb.2014.06.051. 25033437 10.1016/j.colsurfb.2014.06.051

[45] Rebitski EP, Alcântara ACS, Darder M, Cansian RL, Gómez-Hortigüela L, Pergher SBC. Functional carboxymethylcellulose/zein bionanocomposite films based on neomycin supported on sepiolite or montmorillonite clays. ACS Omega. 2018;3:13538–50. 10.1021/acsomega.8b01026. 31458061 10.1021/acsomega.8b01026 PMC6644915

[46] Sandri G, Faccendini A, Longo M, Ruggeri M, Rossi S, Bonferoni MC, et al. Halloysite-and montmorillonite-loaded scaffolds as enhancers of chronic wound healing. Pharmaceutics. 2020;12:179. 10.3390/pharmaceutics12020179. 32093190 10.3390/pharmaceutics12020179 PMC7076487

